# Health equity engineering: Optimizing hope for a new generation of healthcare

**DOI:** 10.1017/cts.2024.549

**Published:** 2024-05-23

**Authors:** Felicity T. Enders, Elizabeth H. Golembiewski, Joyce E. Balls-Berry, Tayla R. Brooks, Allison R. Carr, John P. Cullen, Deborah DiazGranados, Ayorkor Gaba, Leigh Johnson, Terri Menser, Shari Messinger, Adam J. Milam, Minerva A. Orellana, Susan M. Perkins, Tiffany D. Chisholm Pineda, Sally W. Thurston, Vyjeyanthi S. Periyakoil, Alexandra L. Hanlon

**Affiliations:** 1 Department of Quantitative Health Sciences, Mayo Clinic, Rochester, MN, USA; 2 Division of Endocrinology, Diabetes, Metabolism, and Nutrition, Mayo Clinic, Rochester, MN, USA; 3 Department of Neurology, Washington University School of Medicine, St. Louis. MO, USA; 4 Center for Clinical and Translational Science, Mayo Clinic Graduate School of Biomedical Sciences, Rochester, MN, USA; 5 Clinical and Translational Science Institute, Center for Community Health and Prevention and Health Humanities and Bioethics, University of Rochester Medical Center, Rochester, NY, USA; 6 School of Medicine, Virginia Commonwealth University, Richmond, VA, USA; 7 Department of Counseling & Clinical Psychology, Teachers College, Columbia University, New York, NY, USA; 8 Clinical & Translational Science Institute, New York University Langone Health, New York, NY, USA; 9 Division of Health Care Delivery, Mayo Clinic, Jacksonville, FL, USA; 10 Department of Public Health Sciences, University of Miami Miller School of Medicine, Miami, FL, USA; 11 Department of Anesthesiology and Perioperative Medicine, Mayo Clinic, Rochester, MN, USA; 12 Department of Obstetrics and Gynecology, University of Washington, Seattle, WA, USA; 13 Department of Biostatistics and Health Data Science, Indiana University School of Medicine, Indianapolis, IN, USA; 14 College of Journalism, University of Florida, Gainesville, FL, USA; 15 Department of Biostatistics and Computational Biology, University of Rochester Medical Center, Rochester, NY, USA; 16 Department of Medicine, Stanford University School of Medicine, Palo Alto, CA, USA; 17 Department of Statistics, Virginia Tech, Blacksburg, VA, USA

**Keywords:** Health equity, health disparities, chronic stress, accelerated aging, population health

## Abstract

Medical researchers are increasingly prioritizing the inclusion of underserved communities in clinical studies. However, mere inclusion is not enough. People from underserved communities frequently experience chronic stress that may lead to accelerated biological aging and early morbidity and mortality. It is our hope and intent that the medical community come together to engineer improved health outcomes for vulnerable populations. Here, we introduce Health Equity Engineering (HEE), a comprehensive scientific framework to guide research on the development of tools to identify individuals at risk of poor health outcomes due to chronic stress, the integration of these tools within existing healthcare system infrastructures, and a robust assessment of their effectiveness and sustainability. HEE is anchored in the premise that strategic intervention at the individual level, tailored to the needs of the most at-risk people, can pave the way for achieving equitable health standards at a broader population level. HEE provides a scientific framework guiding health equity research to equip the medical community with a robust set of tools to enhance health equity for current and future generations.

## Introduction

Imagine a future scenario in which a 52-year-old patient from a low-income, minoritized community visits their physician. Despite a life marked by chronic stress from racial discrimination and financial hardship, this individual outwardly appears healthy, likely due to a hard-won resilience that masks stress-induced changes at the biological level. During this office visit, a comprehensive screening – perhaps a survey or blood test, affordable or even free for the patient – uncovers early signs of accelerated aging. Prompted by these results, the physician orders additional, targeted testing to pinpoint specific health concerns. At a subsequent follow-up visit, the physician and patient discuss interventions, guided by recent evidence on treatments shown to preempt the onset of chronic disease. Information from the patient’s case seamlessly feeds into a growing repository of anonymized data for further research.

In this scenario from a hypothetical healthcare landscape of the future, there are many benefits: The patient receives earlier disease detection and treatment options; the provider leverages more precise and effective diagnostic tools, justifying the investment in further testing and additional clinical time to help this at-risk patient; the payer realizes cost savings from earlier intervention; and researchers benefit from dynamic, real-world data on chronic stress and the effectiveness of various mitigation strategies. Crucially, society benefits from lower overall morbidity and mortality, particularly in historically marginalized communities, helping to disrupt cycles of generational poverty.

To realize this future, we propose “health equity engineering” (HEE), a novel approach to proactively engineer improvements at the individual level to drive population health equity. Specifically, HEE targets chronic stress and its contribution to premature biological aging, acknowledging the disproportionate impact of stress on minoritized communities and its role as both a cause and effect of health disparities. As a scientific framework, HEE represents a strategic, systemic shift toward achieving comprehensive health equity, utilizing learning health system principles to prompt real-time, actionable improvements in both clinical care and medical research.

**Figure 1.**
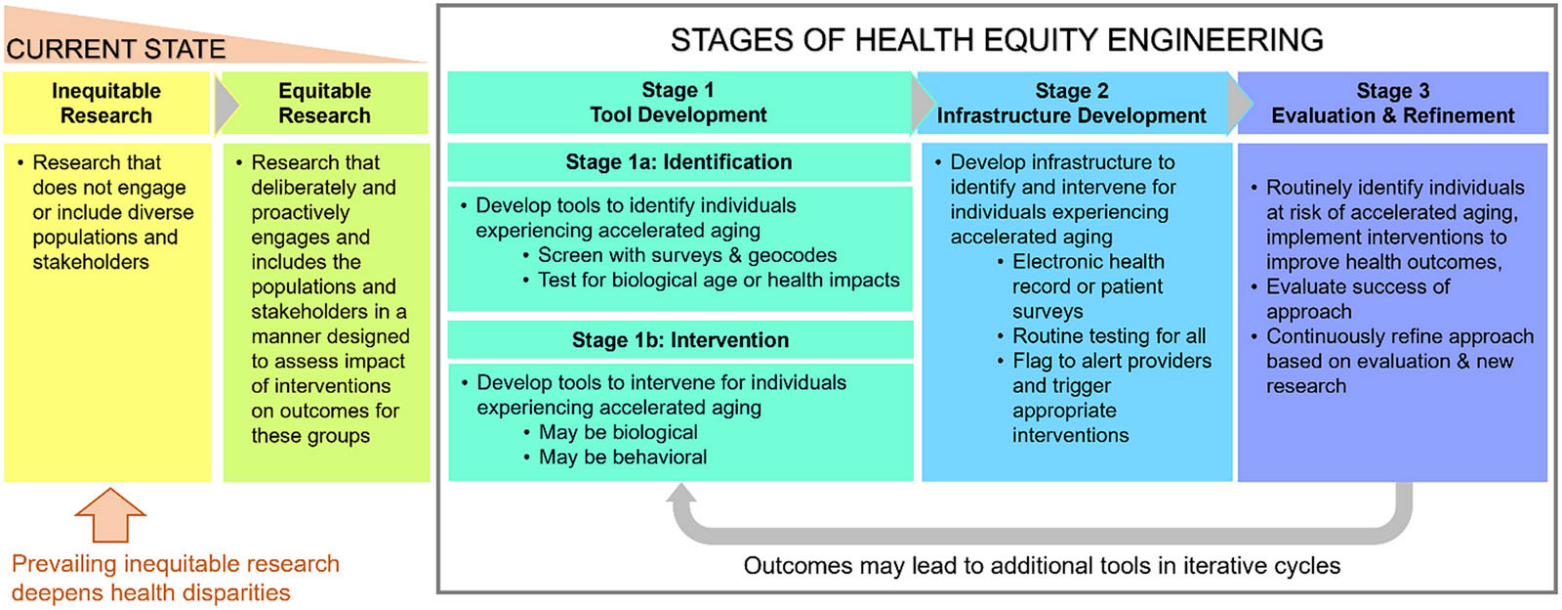
Current research and the proposed stages for research in Health Equity Engineering.

HEE has the potential to transform both clinical care and medical research by advancing health equity across diverse populations. By identifying chronic stress and its clinical sequelae as a primary focus, HEE research will develop and apply practical tools to assess the risk of accelerated aging and offer sustainable, evidence-based interventions tailored to individual needs. While some such tools are currently available, the HEE scientific framework is intended to guide research to develop, test, and refine additional tools. With this comprehensive but pragmatic framework, we aim to guide medicine toward a cohesive and actionable vision of equitable healthcare. Figure 1 contrasts the current state of inequitable research and practice with the proposed stages for HEE research to modify patient care intentionally and inclusively within healthcare systems.

## Current state: Prevailing inequitable research

The need for HEE stems in part from inequitable research practices that contribute to disparities in health status and clinical care. Specifically, medical research often fails to include enough participants from communities disproportionately impacted by chronic stress and its sequelae. In turn, clinical advancements predominantly benefit patients from groups with adequate representation, deepening inequities. This lack of representation also raises uncertainties about the applicability of scientific advances to underserved communities. While some strides have been made to improve inclusion, such as federal requirements for inclusive enrollment in clinical trials and community-engaged research efforts, adequate representation has yet to be fully realized.

The example of women, who historically comprised an underrepresented population in medical research, underscores how intentional actions at the policy and practice levels can drive health equity. In 1985, a pivotal national report recommended inclusion of women across all research to overcome marginalization. Following this, the National Institutes of Health established a 1986 policy mandating the inclusion of women in research, and in 1989, began requiring applicants to justify any planned exclusion of women from studies. Further reinforcing this policy, a 1993 Congressional passed legislation mandating the inclusion of women and individuals from racial and ethnic minority populations in trials such that assessment of impact is possible for these groups, explicitly stating that cost cannot justify exclusion. Progressively, these policies improved widespread inclusion of women as participants in federally funded research, increasing its generalizability and applicability [1].

However, the same level of impact has not been realized for other historically underserved groups. Reasons for ongoing lack of inclusion have been documented as (ironically) cost, inappropriate exclusion criteria, healthcare provider attitudes, sociocultural barriers, access issues, smaller sample sizes, and lack of diversity among research personnel [2]. Additionally, the 1993 law’s specific focus on “trials” has meant that inclusion has lagged even further for other types of studies.

Consequently, decades of underrepresentation in biomedical research have created significant knowledge gaps about the specific health needs and responses to treatments among underserved groups. Thus, even if equitable research was achieved now, it would only improve equity for future scientific advances without reversing decades of historical exclusions. Addressing these injustices will require not only including these groups in research but also addressing the biological mechanisms contributing to health disparities, which we detail in the next section. It is important to note that biological differences are a downstream effect of social, environmental, and political inequities – not innate differences based on race, ethnicity, or other personal characteristics.

## Chronic stress and accelerated aging

Chronic elevated stress, a key issue at the heart of HEE, is more prevalent among certain marginalized communities [3]. As such, patients from these groups often experience increased risk of poor outcomes while simultaneously having fewer tools for treatment than majority groups.

Chronic stress can lead to accelerated biological aging [4,5], affecting cellular processes like DNA methylation [6,7], telomere shortening [8,9], cellular senescence [8,10], and inflammation [11,12]. In turn, these cellular changes contribute to increased incidence of multiple chronic conditions, including hypertension, heart disease, diabetes, stroke, kidney failure, and cancer [13–16], which are well-documented causes of early morbidity and mortality in minoritized populations.

**Figure 2.**
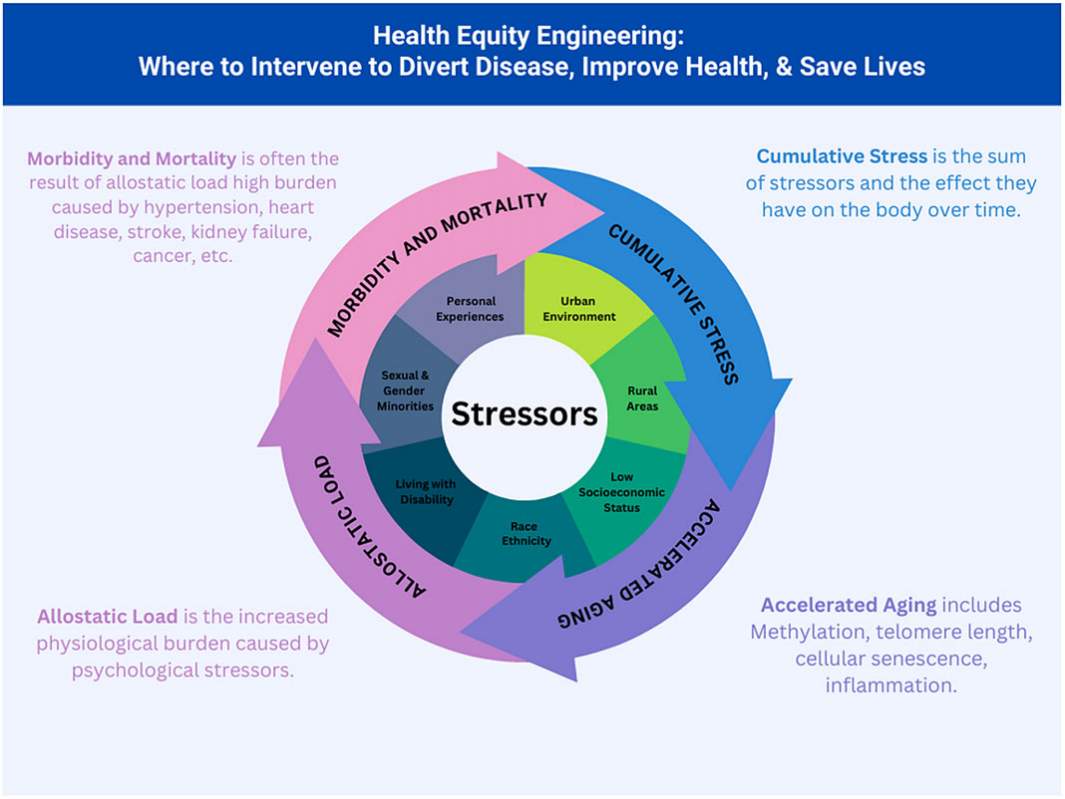
Health Equity Engineering cyclical diagram.

Figure 2 illustrates how compounded stressors accelerate biological aging and elevate allostatic load, heightening the risk of morbidity and mortality in groups with excess chronic stress. In addition, these challenges can perpetuate generational cycles of stress. The ultimate goal of HEE is to disrupt this cycle, improving health outcomes across the lifespan.

### Populations at risk of chronic stress and accelerated aging

Understanding how chronic elevated stress leads to accelerated aging begins with recognizing the groups most at risk based on available evidence (Fig. 2).

### Urban or rural residence

In densely populated inner-city urban settings, stress is intensified by demanding social dynamics, inequality, and environmental factors like overcrowding, pollution, and noise [17]. This stress manifests in higher morbidity and mortality rates and has been shown to accelerate epigenetic aging [18]. At the other end of the continuum, residents of the most rural, isolated areas face similarly heightened risks of stress due to factors like economic challenges and limited healthcare access [19,20].

### Low socioeconomic status (SES)

Generational wealth and generational poverty are structurally embedded within the United States. A key aspect of generational wealth is passing property from one generation to the next. Property taxes are utilized to provide local funding for public schools and, in turn, education is a well-recognized driver of both lifetime earnings and health. As such, SES is intricately linked to health disparities, as the financial and social instability inherent to lower SES manifests as increased stress and higher rates of chronic diseases [21,22]. In turn, ongoing stress activates inflammatory responses in the body [23–25], resulting in higher morbidity rates both directly through accelerated biological aging and indirectly through coping behaviors linked to poor outcomes [26,27]. Compounding these risk factors are the significant disparities in access to resources between individuals with lower vs. higher SES, such as healthcare, safe environments, access to nutritious food, and support systems.

### Race and ethnicity

Increased epigenetic aging has been observed in minoritized racial and ethnic groups. For example, Black individuals exhibit an older epigenetic age than White individuals of the same chronological age; this disparity has been linked to increased cumulative stress and traumatic events among Black communities in the USA [28]. The weathering hypothesis suggests that excess stress is a driver of early morbidity and mortality in those of minoritized race and ethnicity, especially Black people [29–33].

### Living with disability

Individuals living with disabilities constitute a diverse group with varied cognitive, physical, visual, and auditory challenges [34,35]. Individuals with a disability are more likely than others to be in poor health [36], potentially exacerbating stress levels. Although direct evidence linking chronic stress to health outcomes in disabled populations is scarce, the heightened prevalence of chronic stress within these communities is well-documented.

### Sexual and gender minorities

Sexual and gender minorities, encompassing those who do not identify as cisgender heterosexual, face stressors related to societal discrimination, legal challenges, and lack of acceptance in many areas of the country [37,38]. However, lack of comprehensive data on sexual and gender minority groups further complicates understanding of their unique health needs.

### Personal experiences

Anyone can have chronic stress due to personal experiences, including but not limited to food or housing insecurity, living far from work or school, lack of interpersonal support, and inadequate healthcare access. For this reason, our HEE framework emphasizes the need to assess all people for stress and accelerated aging.

### Summary of populations at risk

HEE aims to lessen health disparities for at-risk populations by providing a scientific framework guiding research to develop and implement personalized interventions to intentionally mitigate the health impacts of chronic stress within medical practice. The following sections detail the stages of HEE across both research and practice and offer initial recommendations for its implementation.

## Stage 1: Tool development

In Stage 1 of HEE research, the focus is on developing screening and diagnostic tools to identify individuals who are experiencing or who are at risk of accelerated aging and developing interventions to improve their health outcomes. To set the stage for proposing research on how we as a research community might develop such tests, we begin by discussing implications for practice on who should be tested and when testing should be initiated.

## Stage 1a. Identifying individuals: Who, when, and how to test

We recommend that medical professionals assess biological aging *for all people* to identify those at risk of health issues due to chronic stress. Early identification in clinical settings will enable timely interventions for conditions associated with accelerated aging, which might otherwise go unnoticed.

### Who to test

Patients from all backgrounds should be screened for signs of accelerated biological aging and/or excessive lifetime exposure to stress. This approach recognizes that while certain groups are known to be at higher risk due to chronic stress, individuals outside these groups may also be affected [39]. Testing only individuals from specific groups would create disparity both by equating accelerated aging with demographic characteristics and by missing individuals with chronic stress outside these groups. Furthermore, as socio-cultural norms, so too may the groups most at risk, necessitating an inclusive testing strategy.

### When to test

Ideally, testing for accelerated aging will be accessible, noninvasive, and free for everyone. We recommend that testing takes place early in life to allow for earlier intervention. Testing for stress exposure when individuals turn 40 years of age may be particularly beneficial, as research has shown that chronic disease incidences begin to diverge across different racial and ethnic groups around this age [40–42]. However, the optimal age range for testing may vary for different disease processes and should be adjusted as our understanding of health equity evolves.

### How to test

In this section, we describe the research needed to develop tools for Stage 1a of HEE. Given the logistical challenges, cost, and lack of consensus currently associated with direct measurement of biological age at the individual level [43–48], HEE will require more accessible means of initial screening for lifetime stress exposure as the research on direct testing evolves. These may include assessments based on survey measures and/or geocoded data, the findings of which may signal the need for more extensive, targeted testing for individual patients. We acknowledge the need for a thorough evaluation of existing measures to ensure their relevance across diverse populations.

Various survey instruments have been developed to capture lifetime stress based on specific experiences, such as the Perceived Stress Scale (PSS), the Stress and Adversity Inventory (STRAIN), the Adverse Childhood Experiences Scale (ACES), and others (Table 1). However, we argue that the ideal screening survey does not yet exist. An HEE screening tool should include three critical attributes. First, it must be brief, so patients in all settings can feasibly complete it. While ACES is brief, it only addresses distressing events experienced in childhood. Critically, an HEE screening tool should assess *life course* experiences to align with accelerated aging from lifetime stress. We found several surveys that covered the life course, most notably the Life Stressor Checklist-Revised, the Experiences of Discrimination Scale (EOD), and STRAIN. Of these, only the EOD can be considered brief (nine items). Finally, HEE screening should consider generalized stress from *any* potential source, rather than focusing only on specific experiences. The PSS is brief and considers generalized stress rather than specific experiences, but the timeframe covered is only the prior month. This highlights a critical future need for HEE implementation: a brief survey measure that can comprehensively measure lifetime and generalized stress.

**Table 1.** Existing survey measures to screen for accelerated biological aging

Year	Measure	Number of Items	Recall Period	Reference
1983	Perceived Stress Scale (PSS)	14	Past month	Cohen S, Kamarck T, Mermelstein R. A global measure of perceived stress. *J Health Soc Behav*. 1983;24(4):385–96.
1996	Index of Race-Related Stress (IRRS)^a,b^	46	Lifetime	Utsey SO, Ponterotto JG. Development and validation of the Index of Race-Related Stress (IRRS). *J Couns Psychol*. 1996;43:490–501.
1996	Perceived Racism Scale (PRS)	51	Past year; Lifetime	McNeilly MD, Anderson NB, Armstead CA, *et al.* The Perceived Racism Scale: a multidimensional assessment of the experience of white racism among African Americans. *Ethn Dis*. 1996;6(1-2):154–66.
1996	Perceptions of Racism Scale (TPRS)	20	No explicit recall period	Green NL. Development of the Perceptions of Racism Scale. *J Nurs Sch.* 1995;27(2):141–16.
1996	Schedule of Racist Events (SRE)	18	Past year; Lifetime	Landrine H, Klonoff EA. The Schedule of Racist Events: a measure of racial discrimination and a study of its negative physical and mental health consequences. *J Black Psychol.* 1996;22(2):144–168.
1997	Everyday Discrimination Scale (EDS)^a^	9	No explicit recall period	Williams DR, Yan Yu, Jackson JS, Anderson NB. Racial differences in physical and mental health: socioeconomic status, stress and discrimination. *J Health Psychol.* 1997;2(3):335–51.
1997	Life Stressor Checklist-Revised (LSC-R)	30	Lifetime	Wolfe J, Kimerling R, Brown P, *et al.* The Life Stressor Checklist-Revised (LSC-R) [Measurement instrument]. 1997. Available from http://www.ptsd.va.gov.
1998	Adverse Childhood Experiences Scale (ACES)	10	Childhood	Felitti VJ, Anda RF, Nordenberg D, *et al.* Relationship of childhood abuse and household dysfunction to many of the leading causes of death in adults: the Adverse Childhood Experiences (ACE) Study. *Am J Prev Med*. 1998;14(4):245–58.
2000	Adolescent Discrimination Distress Index (ADDI)	15	Lifetime	Fisher CB, Wallace SA, Fenton RE. Discrimination distress during adolescence. *J Youth Adolesc.* 2000;29(6):679–95.
2001	Perceived Ethnic Discrimination Questionnaire (PEDQ)	22	Lifetime	Contrada RJ, Ashmore RD, Gary ML, *et al.* Measures of ethnicity-related stress: psychometric properties, ethnic group differences, and associations with well-being. *J Appl Soc Psychol.* 2001;31(9):1775–820.
2005	Perceived Ethnic Discrimination Questionnaire-Community Version (PEDQ-CV)	22	Lifetime	Brondolo E, Kelly KP, Coakley V, *et al.* The Perceived Ethnic Discrimination Questionnaire: development and preliminary validation of a community version 1. *J Appl Soc Psychol*. 2005;35(2):335–65.
2004	Asian American Racism-Related Stress Inventory (AARRSI)	29	Lifetime	Liang CT, Li LC, Kim BS. The Asian American Racism-related Stress Inventory: development, factor analysis, reliability, and validity. *J Couns Psychol*. 2004;51(1):103.
2005	Experiences of Discrimination Scale (EOD)	9	Lifetime	Krieger N, Smith K, Naishadham D, *et al.* Experiences of discrimination: validity and reliability of a self-report measure for population health research on racism and health. *Soc Sci Med*. 2005;61(7):1576–96.
2008	Measure of Indigenous Racism Experiences (MIRE)	31	No explicit recall period	Paradies YC, Cunningham J. Development and validation of the Measure of Indigenous Racism Experiences (MIRE). *Int J Equity Health*. 2008 Apr 22;7:9.
2018	Stress and Adversity Inventory (STRAIN)	20	Lifetime	Slavich GM, Shields GS. Assessing lifetime stress exposure using the Stress and Adversity Inventory for Adults (Adult STRAIN): An overview and initial validation. *Psychosom Med*. 2018;80(1):17–27.
2017	Comprehensive Score for Financial Toxicity (COST)	12	Past 7 days	de Souza JA, Yap BJ, Wroblewski K, *et al.* Measuring financial toxicity as a clinically relevant patient-reported outcome: the validation of the COmprehensive Score for financial Toxicity (COST). *Cancer*. 2017 Feb 1;123(3):476–484.
2022	Survey of Consumer Finances (SCF)	Varies	Triennial survey	Board of Governors of the Federal Reserve System. 2022 Survey of Consumer Finances (SCF). https://www.federalreserve.gov/econres/scfindex.htm

a Brief versions available.

b Adolescent version available.

An additional approach to assessing life course stress involves the use of geocoded data, which can offer estimates of stress levels based on location-specific societal and environmental factors. While this method cannot capture all aspects of personal stress or individualized stress responses and necessitates regular updates due to the dynamic nature of environmental stressors, geocoded data provide significant insights into stress influenced by location (see Table 2) with the added advantage of limiting survey fatigue for patients. Future geocoded measures could account for lifetime stress risk by assessing specific times lived at different addresses. As geocoded assessment in statistical software is a relatively recent advance, we anticipate significant growth in research on geocoded assessment of environmental stressors over time.

**Table 2.** Geocoded and geographically based indicators of stress

Name	Data Source	Geographic Unit	Description	Items	Reference
Area Deprivation Index (ADI)	Secondary data	Census block group	Ranked measure of neighborhood relative socioeconomic disadvantage based on income, education, employment, and housing quality	17	Kind AJH, Buckingham WR. Making neighborhood-disadvantage metrics accessible: the Neighborhood Atlas. *N Engl J Med*. 2018;378(26):2456–2458.
Collective Efficacy Scale (CES)	Individual self-report	Neighborhood	Extent to which an individual believes their neighbors work together using two subsections: 1) informal social control (how likely neighbors are to intervene when there is trouble), and 2) social cohesion and trust (how likely neighbors are to support each other in times of need)	10	Sampson RJ, Raudenbush SW, Earls F. Neighborhoods and violent crime: a multilevel study of collective efficacy. Science. 1997;277(5328):918–24.
Community Disadvantage Index	Secondary data	Census tract	Neighborhood disadvantage as quantified by rates of educational attainment, home ownership, poverty, and single-parent households	4	Ross CE, Mirowsky J. Neighborhood disadvantage, disorder, and health. *J Health Soc Behav.* 2001;42(3):258–76.
County Structural Racism (CSR) Index	Secondary data	County	Structural racism based on indicators of education, housing, employment, criminal justice, and health care access	5	Dougherty GB, Golden SH, Gross AL, *et al.* Measuring structural racism and its association with BMI. *Am J Prev Med*. 2020;59(4):530–537.
HOUsing-based index of SocioEconomic Status (HOUSES)	Secondary data	Housing unit	Derived from address-linked, publically available data on property value, square footage, and number of bedrooms and bathrooms associated with a housing unit	4	Juhn YJ, Beebe TJ, Finnie DM, *et al.* Development and initial testing of a new socioeconomic status measure based on housing data. *J Urban Health*. 2011;88(5):933–944.
Index of Concentration at the Extremes (ICE)	Secondary data	Various	Economic and social polarization within a defined geographic area (i.e., how concentrated a population is in extremes of deprivation and privilege)	2	Massey DS. The prodigal paradigm returns: ecology comes back to sociology. *Does It Take Village.* 2001:41–8.
Medically Underserved Areas/Populations (MUA/P)	Secondary data	Various	Federal designation for geographic areas with a lack of access to primary health care services or populations within a defined geographic area facing economic, cultural, or linguistic barriers to health care (e.g., people experiencing homelessness, low-income, migrant farmworkers)	4	https://bhw.hrsa.gov/workforce-shortage-areas/shortage-designation
Multidimensional Measure of Structural Racism (MMSR)	Secondary data	Public Use Microdata Areas (PUMAs)	Structural racism as measured by residential segregation and inequities in education, employment, income, wealth, and incarceration	6	Chantarat T, Van Riper DC, Hardeman RR. The intricacy of structural racism measurement: A pilot development of a latent-class multidimensional measure. *EClinicalMedicine*. 2021;40:101092.
Neighborhood Inventory for Environmental Typology (NIfETy)	Observer rating	Neighborhood	Measures the prevalence of environmental factors that may be linked to youth exposure to alcohol, tobacco, violence, and other drugs	129	Furr-Holden CD, Smart MJ, Pokorni JL, *et al.* The NIfETy method for environmental assessment of neighborhood-level indicators of violence, alcohol, and other drug exposure. *Prev Sci.* 2008;9(4):245–55.
Neighborhood Environment Scale (NES)	Individual self-report	Neighborhood	Perceived violence, safety, drug use and availability of drugs in the neighborhood	18	Elliott DS, Huizinga D, Ageton SS. *Explaining delinquency and drug use.* Sage Publications; Beverly Hills, CA: 1985.

## Stage 1b. Interventions to improve outcomes

Following identification of at-risk individuals, another component of HEE Stage 1 is research to develop interventions and treatments to mitigate or reverse the health impacts of accelerated aging. Fortunately, HEE Stages 1a and 1b can happen simultaneously. A brief overview of existing tools is broadly categorized here into behavioral, interpersonal, and biological interventions.

### Behavioral interventions

Lifestyle interventions focusing on fasting, nutrition, exercise, and sleep are instrumental in reducing inflammation and improving overall health [49–51]. In particular, fasting has been shown to initiate autophagy [52], in which the body breaks down damaged cellular components. HEE also aims to promote resilience to mitigate the impact of stress on accelerated aging. Resilience interventions, including those promoting mindfulness, psychoeducation, and social support, have demonstrated positive effects on mental health and cardiovascular health [53,54]. Integrating mental health strategies with lifestyle changes will be key for long-term health benefits under an HEE approach. Structural changes may also be required to make these individual-level interventions more accessible and effective for those most susceptible to chronic stress.

Research will be required to guide providers on how to effectively introduce behavioral interventions to patients. We envision communication tools to frame the patient-provider discussion around feasible interventions tailored to the patient’s needs. In order to avoid patronizing language, communication tools should actively recognize that the patient and provider are partnering to overcome impacts attributable to a host of circumstances at the personal, interpersonal, institutional, and societal levels, with visualization based on the social-ecological framework [55]. This approach emphasizes that the provider understands that the patient may be experiencing stress from multiple sources that are not under their control. Discussions should emphasize that the patient still has power over their own future and that the provider is there to help with tools designed for this purpose.

### Interpersonal interventions

Within healthcare systems, HEE will target clinician behaviors to improve health equity. Misconceptions about racial and ethnic differences, along with unconscious biases among healthcare professionals [56], contribute significantly to health disparities [57–59]. Addressing these through diversity, equity, and inclusion initiatives, such as unconscious bias assessments and cultural competency training, is essential to counteract biased behavior toward patients. Comprehensive patient management by primary care and targeted interventions by specialists are vital components of HEE.

### Biological interventions

Recent advances in aging research have led to new tools that show promise in reversing accelerated aging and preventing age-related diseases. In particular, novel senolytic drugs offer a groundbreaking avenue to reverse the accelerated aging process through elimination of senescent cells that contribute to aging and age-related diseases [60–64]. The emergence of these therapeutics can potentially pave the way for a future where the rejuvenation of aging tissues becomes an achievable reality. As clinical trials and research in this field progress, senolytic drugs may offer transformative solutions for extending the human lifespan and alleviating the burdens of accelerated aging.

Cellular mechanisms for autophagy play a crucial role in preventing senescence. As with many cellular processes, the efficiency of autophagy declines with age, allowing for the buildup of dysfunctional cellular materials and contributing to senescence. In addition to fasting, several available drugs, such as rapamycin and metformin, are known to induce autophagy and, like senolytic drugs, may provide a pathway to prevent or even reverse cellular processes associated with aging or senescent cells. Notably, their established safety profiles and availability as generic medications increase their potential for widespread use in combating age-related cellular changes.

## Stage 2: Infrastructure development

In Stage 2, the focus of HEE research will be on informatics to integrate the tools identified or developed in Stage 1 into healthcare systems. These tools should be thoroughly tested and then incorporated into clinical workflows, ensuring that responses prompt appropriate referrals or other actions necessary for personalized patient care. Successful implementation will also require provider buy-in, facilitated by applying learning health system principles and encouraging collaboration between researchers, clinical providers, and engineers. In addition, sustainability of HEE interventions will rely on scalable approaches that can adapt to dynamic healthcare environments. Additional sustainability measures include ongoing training and support for the use of new tools and practices and continuous monitoring and refinement of tools to maximize their impact.

A critical component of Stage 2 is the use of electronic health record (EHR) software to broadly implement HEE initiatives. Initially, institutions will focus on developing and refining HEE strategies within learning health system cycles. Once optimized, integrating these strategies into EHR systems will enable rapid dissemination of best practices, eliminating the need for separate support infrastructures among different institutions and thereby streamlining the implementation process. When a patient is identified, it is essential that the system flag the patient for further follow-up by alerting the provider.

## Stage 3: Implementation and evaluation

The work of HEE will need to continue beyond the development and implementation of tools to identify and intervene for at-risk patients in healthcare systems. Specifically, continuous evaluation will be necessary to identify ongoing opportunities to improve health outcomes, creating a dynamic cycle of developing and implementing new tools for health optimization. This will require a learning health system approach to continually fine-tune existing tools and identify patient-centered gaps [65].

### Immediate implementation

While we have introduced stages for HEE research, medical practitioners and researchers can begin to implement HEE immediately. For medical practice, one can utilize the current tools (Tables 1 and 2) to help identify people at risk of chronic stress and accelerated aging and follow with more frequent testing. Although we lack an ideal screening tool for lifetime stress, ACES can effectively identify significant stress quickly and can be implemented to begin HEE work immediately. Implementation in medical practice might be carried out within a given healthcare system or in a specific field prior to broad adoption. A key consideration for HEE implementation will be encouraging behavior change among at-risk persons in a respectful and non-paternalistic manner. To effectively engage patients in conversations about behavioral modifications, providers can practice empathetic engagement and motivational interviewing, skills that may necessitate additional training or education.

One critical caution for immediate implementation is a potential unintended consequence of HEE. We are concerned that a new focus on accelerated aging could actually increase disparity. That would occur if testing for accelerated aging was instigated only for high-resource patients. However, the HEE framework is designed to drive research and implementation specifically to forestall that uncomfortable outcome.

Conversely, immediate HEE implementation may deter a different source of disparity. As healthcare increasingly integrates artificial intelligence and digital technologies, there is a risk that health disparities will be exacerbated for populations already underrepresented in digital health advancements. HEE sidesteps this concern by providing a framework to leverage widespread testing and digital data to bridge these gaps, ensuring that all communities benefit from technological progress in healthcare.

### Future research

For implementing HEE research, we have highlighted numerous avenues for new and focused action across various HEE components. We envision that this research will unlock new possibilities across multiple arenas with the overall goal of changing healthcare to improve equity through individual identification and intervention. Specifically, HEE research will require input from a wide range of disciplines, including psychometricians, statisticians, and qualitative researchers (Stage 1a); psychologists, behavioral scientists, communication experts, and implementation scientists (Stage 1b, Behavioral Interventions); bench scientists, translational scientists, and aging researchers (Stage 1b, Biological Interventions); informaticists, learning health system researchers, and implementation scientists (Stage 2); and clinicians and scientists (Stage 3).

Central to the HEE framework is ongoing research into the relationship between chronic stress and accelerated biological aging and identification of effective strategies to enhance health outcomes for at-risk patient populations. Key avenues for future research into the former include clarifying the dose-response relationship between stress and accelerated aging, including the threshold at which stress begins to impact biological age and opportunities for prevention; the differential impact of stress experienced earlier vs. later in life; and the role of resilience in mitigating the impacts of stress. Answering these and related questions will be important to refine measures used for estimating, examining, and intervening in lifetime exposure to stress. HEE research will also further evidence on the most effective approaches for screening and treatment, including the optimal age for screening, as well as strategies for its implementation among populations with limited or no access to healthcare. In particular, screenings such as colonoscopies and mammograms might be even more effective if timed based on biological age rather than chronological age.

Future research also holds promise for building community-research partnerships. By engaging with marginalized groups, HEE opens a door to engaging with communities that have historical distrust of medical professionals. Discussions of HEE can begin by acknowledging mistakes made by the medical community at large, a step which in our experience is not frequently taken. Furthermore, HEE includes specifics of how to help people, with an explicit focus on helping those most at risk of poor outcomes. As such, we foresee HEE acting as a catalyst for more inclusive research practices with the potential for greater representation of marginalized populations in health research.

## Conclusion

HEE introduces a systemic, multisector approach to mitigate the effects of health disparities. Moving beyond the specialization-focused nature of modern medicine [66,67], HEE adopts a holistic approach that recognizes the impacts of accelerated biological aging on the entire body and represents a new dimension of personalized medicine that intentionally addresses the needs of historically marginalized and underserved populations. HEE provides a scientific framework guiding health equity research to equip the medical community with a robust set of tools as well as a framework for broader application, aiming to enhance health equity for current and future generations.

This paper was developed by the Justice, Equity, Diversity, and Inclusion special interest group of the Association for Clinical and Translational Science. We share the HEE framework as part of our ongoing efforts to advance health equity and diversity, equity, and inclusion in medical research. Notably, 14 of the 18 authors are members of one or more populations at risk for accelerated aging as described in this paper. We believe the impacts of health disparities can be substantially diminished or even reversed through combined efforts from across the medical research enterprise. We believe health equity can be engineered by working at the individual level to intervene and improve outcomes at the population level.
